# Energy Efficient Strategy for Throughput Improvement in Wireless Sensor Networks

**DOI:** 10.3390/s150202473

**Published:** 2015-01-23

**Authors:** Sohail Jabbar, Abid Ali Minhas, Muhammad Imran, Shehzad Khalid, Kashif Saleem

**Affiliations:** 1 Department of Computer Science, Bahria University Islamabad, Islamabad 44000, Pakistan; E-Mails: abid.research@gmail.com (A.A.M.); shehzad_khalid@hotmail.com (S.K.); 2 King Saud University, P.O. Box 92144, Riyadh 11543, Saudi Arabia; E-Mails: cimran@ksu.edu.sa (M.I.); ksaleem@ksu.edu.sa (K.S.)

**Keywords:** wireless sensor networks, network lifetime, clustering, energy-aware routing, throughput maximization

## Abstract

Network lifetime and throughput are one of the prime concerns while designing routing protocols for wireless sensor networks (WSNs). However, most of the existing schemes are either geared towards prolonging network lifetime or improving throughput. This paper presents an energy efficient routing scheme for throughput improvement in WSN. The proposed scheme exploits multilayer cluster design for energy efficient forwarding node selection, cluster heads rotation and both inter- and intra-cluster routing. To improve throughput, we rotate the role of cluster head among various nodes based on two threshold levels which reduces the number of dropped packets. We conducted simulations in the NS2 simulator to validate the performance of the proposed scheme. Simulation results demonstrate the performance efficiency of the proposed scheme in terms of various metrics compared to similar approaches published in the literature.

## 1. Motivation

Synergistic mating of wireless communication, sensing and network technology paves the way for the emergence of wireless sensor networks (WSN) [1] that open an enormous range of applications in various domains such as surveillance, tracking, healthcare, and in environmental science [2]. Most of these applications require unattended sensors with non-renewable scarce energy resources to stay operational for a longer period of time. The successful operation of WSN in these applications primarily relies on routing sensed data from sensor nodes to the Base Station (BS). Sensor nodes dissipate most of their energy in routing which limits network lifetime. Therefore, routing protocols must be designed in such a way that minimizes energy consumption and extends network lifetime. Direct communication from sensors to the BS is only feasible for very small WSNs and network size is the function of maximum communication range of nodes. For large scale networks, multi-hop communication provides scalability through the transit nodes' to destine the data to far distantly placed BS. Simulation experiments have shown the effectiveness of multi-hop communication over direct in [3].

**Figure 1.**
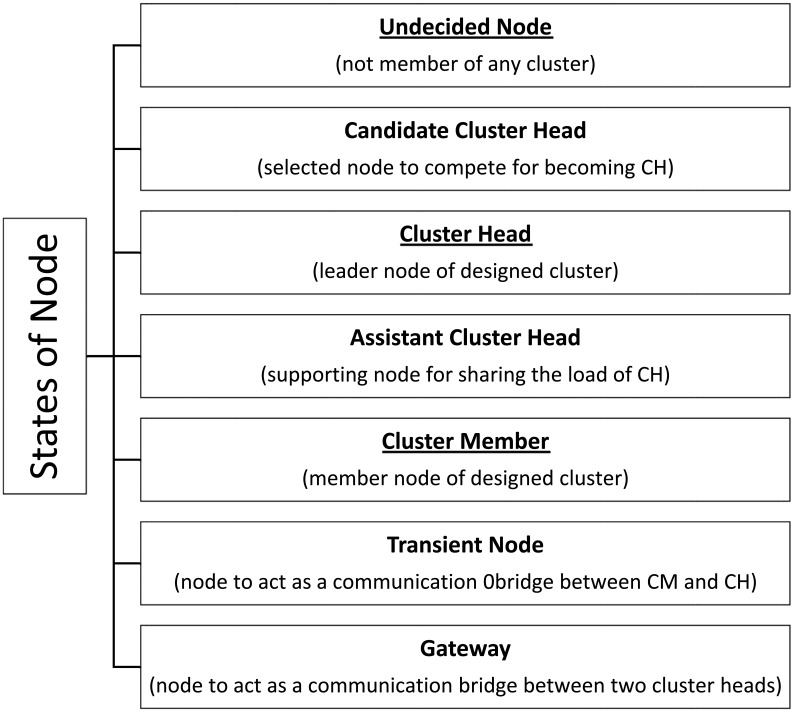
The various roles of nodes during their lifetime in a clustered network architecture.

For minimum utilization of battery energy, all the steps from node deployment to network architecture (flat/clustered) and from environment sensing to communicating the sensed data to the BS (routing) should be carefully designed. In a clustered network architecture [4], nodes are grouped together in clusters with one node called Cluster Head (CH) designated as their head and other nodes in the clusters designated as Cluster Members (CM). In the clustered network, the process of designating a node as the cluster head, *i.e.*, CH election, is usually the initial phase, while establishing the route for communicating the data from the source to the destination is usually its last phase [5], but this sequence is not always the case. Hence, in a clustered network architecture, nodes are designated different roles. Figure 1 shows the possible roles of a node during its lifetime depending upon the underlying clustering algorithm. The node roles that are underlined in the figure are the part of every clustering algorithm. For better understanding of each role, a short description is also given for each one.

Energy efficient routing strives to minimize energy consumption by improving various factors of a number of functional aspects such as communications to the sink, cluster design, CH election, inter-cluster and intra-cluster communication style, and CH rotation. Although a clustered network has priority over a flat network for better network performance in many aspects, its cluster design part is still more energy consuming [6]. Recently, a notable effort to design a multilayer cluster (*i.e.*, MCDA) was reported in [5], where the authors showed that reducing the broadcasting, decreasing computation and packet overhead all contribute to energy conservation. More-over, direct hop communication from source node to sink node as adapted in [7,8] is an energy-aware solution for small networks. This limitation is removed by multi-hop communication style, which also has its number of variants. Some algorithms are just two level multi-hop from source to sink as in [9] and some are pure multi-hop without any limit of hop count from source to sink as in [10]. This last fashion of communication is the most preferable choice among the two options, *i.e.*, direct hop and two level multi-hop. Akhtar *et al.* [11] have presented a hybrid solution to further improve the network efficiency with respect to energy consumption in routing processes. The same hybrid technique is analyzed for its dependability and reliability in intra-cluster routing technique in temperature sending and battlefield applications in [3]. A self-optimizing scheme for energy balanced routing in wireless sensor networks using ants to improve the network performance is comprehensively discussed in [12]. Another approach is using the multipath strategy to improve the network performance by increasing the throughput [13]. A detailed survey of multipath routing and its energy efficient impact on network along with research challenges is explained by Radi *et al.* [14].

In this paper, we have targeted the issue of improving network throughput from the aspect of energy conservation for routing and its related functionalities. Though the proposed scheme can work with any clustered network designing algorithm, here we have exploited the proposed architecture in MCDA since it is a state-of-the-art network architecture. The literature is rich in cluster-based routing protocols that mostly encompasses: (i) cluster design; (ii) route establishment and (iii) CH rotation processes. Cluster design consists of CH selection, cluster member affiliation to cluster heads and time slot assignment for communicating the sensed data to the CH. All the proposed algorithms for the same either accomplish these processes with central control of BS [10] or with a locally controlled distributed style [15]. Both techniques have their pros and cons. The cluster design part from the point of view of the aforementioned three steps is out of the scope of this underlying article. The second (route establishment) and third (CH rotation) steps are covered comprehensively in this article.

In exploiting the architecture of MCDA, we offer suitable algorithms for routing (inter-cluster and intra-cluster) and CH rotation. All nodes in the first tier (first layer) may act as forwarding nodes for the second tier CHs. The cluster heads of each subsequent layer communicate with the CHs of the preceding layer either directly or through intermediate nodes. These intermediate nodes have once acted as decision maker nodes in the election of the CH during the cluster design process. Threshold levels are introduced for the node energy to initiate the process of CH rotation. This role is transferred to the most energy carrying nodes in two steps: load balancing and load transferring. These strategies in collaboration with the MCDAs' architecture work well to provide reliability (safe and stepwise role transfer), fault tolerance (higher density node selection for CHs and for decision maker nodes and thus for the forwarding node), better throughput and improved network lifetime (balanced network utilization, less inter node communication, removing hot spot area in the neighbor of BS, *etc.*). Hence, based on this discussion, we can summarize the contributions of our work as follows:

1. Proposed algorithms, some of whose components have been implemented and evaluated in our previous work [11,16] are used to exploit the MCDA architecture to get the maximum advantages out of a clustered WSN.
2. In a flat network architecture, the information of optimal forwarding nodes among the available forwarding node set is kept in a neighbor table. The proposed strategy *i.e.*, Energy Aware Routing (EAR) has successfully implemented the same idea in clustered networks with suitable customization and modification by considering the energy awareness aspect. Enlisting candidate decision maker nodes in a neighbor table during the cluster design process, introduction of two threshold levels of CH energy are the major ones among these.
3. Same style of Forwarding Node (FN) selection as mentioned in a previous point is subjugated for the CH and FN rotation strategy as well. This helps a good deal in conserving energy and hence in improving the network lifetime.

Rest of the paper is organized as follows: a comprehensive literature survey of the state-of-the-art techniques and comparative analysis is presented in Section 2. Section 3 explains the proposed solution, followed by comparative analysis of the proposed technique with competing algorithms in Section 4. Conclusions and references are given in later sections.

## 2. Literature Survey

In this section, we present some of the state-of-the-art techniques that we have also used, apart from [17], for the comparison with our proposed technique. Also at the end of this section, a tabular representation of various related techniques is given for ease in comparative analysis thereof.

The Multilayer Cluster Designing Algorithm (MCDA) for Lifetime Improvement of wireless sensor networks by Jabbar *et al.* [5] is a hybrid approach in its communication architecture and architectural design perspectives. MCDA uses a multilayer approach comprising a first flat layer in the BS footprint and subsequent clustered layers. The design of former layer is initiated centrally whilst a distributed fashion is applied in the design of the latter. The deployed nodes in the flat layer are termed as first layer nodes. The authors' start the network clustering from the second layer up to the network boundary. The cluster heads in the second layer are selected by the elected decision maker nodes of the first layer. Neighbor Counter, Decision Maker Nodes and Packet Sequence ID with Postfix Counter are the key factors in designing the clusters. Neighbor Counter is used at various steps for selecting one node over others for selection of various roles, *i.e.*, decision maker, cluster head, while Decision Maker Nodes are used for selection of cluster heads from the subsequent layer, and Packet Sequence ID with Postfix Counter is a packet id that is used for grouping nodes and for choosing one node over others from that group for becoming a CH. Second layer nodes elect the node with highest node density as their decision maker node. Second layer nodes communicate their nodal density in their turn to their decision maker nodes to take part in the competition for becoming a CH. Time slots are assigned to these nodes based on the Time Division Multiple Access (TDMA) technique. When the first node of the second layer communicates its nodal density to a decision maker node, it assigns a sequence number with postfix counter “0” to this packet. All the recipient nodes of the second layer nodes save this packet sequence number and become a part of the same group. All the nodes having packets with the same packet sequence number are included in the same group. Only those nodes of a group communicate their nodal density to decision maker nodes which have highest nodal density than their previous nodes. These nodes increment the postfix counter, that provides a twofold advantage: (i) to let the other member nodes of the same group know about their nodal density; (ii) to let the non-member nodes know that they should neither continue this postfix counting nor should they save any info about other group's member nodes. This postfix counter assists the packet sequence number in separating the members of one group from other. The node right after the last member of first group communicates to its decision maker node and assigns a new packet sequence number with postfix counter “0”. After collecting the nodal density of the second layer's selected nodes, the decision maker nodes elect the CH having the highest nodal density among the second layer's addressed nodes. The elected cluster heads broadcast “*Join Request*” packets. This is to inform other sensor nodes of its availability as a CH. Recipient nodes send their consent message in the form of “*join accept*” messages to become the cluster members. If a “*join Request*” message is received from more than one CH then the membership decision is based on the current load on the CH, *i.e.*, the CH having a smaller number of member nodes is preferred to be attached with it.

Another idea by Jabbar *et al.* [16] with the name Threshold Based Load Balancing Protocol for Energy Efficient Routing in WSN (TLPER) exists in the literature. The idea considers nodal density and geographical location of nodes to decide centrally at the BS about the cluster heads and distributed selection of cluster members. Their proposed design involves assistant CHs with Load Balancing Threshold and Role Transfer Threshold techniques. On approaching the first threshold level, a node having the highest energy level in the cluster called assistant cluster head is selected to share the load of the CH. The CH uses this node as its forwarding node rather than directly sending the CH data to the next cluster. An assistant CH either sends this received data directly to the BS or to the next assistant CH of an adjacent cluster. Using the dynamic power adjustment technique, energy utilization in data transmission is saved since the assistant CH is far nearer to the CH compared to the CH of the next cluster. Another idea for introducing assistant clusters was introduced by Wang [17] for power mitigation. The authors name these assistant clusters as partaker nodes. These special nodes assist the CH in the routine job of data collection. Instead of having the CH collect data solely from all sensors in the cluster, a certain number of partaker nodes participate in the data collection. They help collect the raw data, and perform initial data aggregation and necessary processing before transferring data to the CH. With partakers, a portion of power that would have been consumed by CHs is handled by the partakers.

Another proposed mechanism; Energy Aware Distributed Unequal Clustering (EADUC) by Yu *et al.* [18] is an energy-aware routing algorithm for cluster-based wireless sensor networks. They introduced unequal size clusters to remedy the hot spot issue that results in better network lifetime. The designing of the clustering topology comprises a neighbor node information collection phase, cluster head competition phase and cluster formation phase. These constituents make up the setup phase. Each one is given a specific duration, *i.e.*, *T*_1_, *T*_2_ and *T*_3_. Next to it is the data transmission phase. To start the cluster formation process, the BS broadcasts a signal at a certain power level. Each node can compute its approximate distance to the BS based on the received signal strength. In the first phase, each node broadcasts a *Node_Msg* message within its radio range r having node id and its residual energy E_r_. Based on the collected information, each node calculates the average residual energy of its neighbor nodes. The next calculation is of the waiting time *t* using the following Equation (1):

(1) $$t=\{\begin{array}{c} \frac{E_{a}}{E_{r}}T_{2}V_{r},E_{r}\geq E_{a}\\ T_{2}V_{r},E_{r}<E_{a} \end{array}$$

where *V_r_* is a real value randomly distributed in [0.9,1] which is introduced to reduce the probability that two nodes send *Head_Msg*s at the same time. Each node waits for this calculated time prior to broadcasting a *Head_Msg* message.

To start the cluster head selection competition phase, each node compares its average residual energy to its neighbor's calculated average residual energy and decide whether to be a cluster head or not. After waiting for the calculated time *t* the decision of the nodes to be cluster heads is broadcast within their calculated radio range. If a node does not receive any *Head_Msg* message until the expiry of its time *t* then it broadcasts the *Head Msg* within radio range *R_ci_* to advertise that it will be a cluster head.

The competition radius *R_C_* determines the size of the cluster that is based on the proximity to the BS, and each node calculates its own value for *R_C_* using the following Equation (2):

(2) $$R_{C}=\left[1-\alpha \frac{d_{\text{max}}-d\left(s_{i},BS\right)}{d_{\text{max}}-d_{\text{min}}}-\beta \left(1-\frac{E_{r}}{E_{\text{max}}}\right)\right]R_{\text{max}}$$

where *d_max_* and *d_min_* are the maximum and minimum distance from the nodes in the network to the BS, s(*s_i_*, *BS*) is the distance from node *s_i_* to the BS, α and β are weighted factors having values in [0,1], and $R_{c}^{0}$ is the maximum value of the competition radius. This makes the cluster size bigger for a farther elected cluster head and smaller for a nearer elected cluster head. Each non-cluster-head node chooses the nearest cluster head and sends the Join_Msg which contains the id and residual energy of this node. The authors also modified this technique for heterogeneous networks by introducing an energy factor in the radio range competition radius in order to maximally exploit the higher energy nodes. A cluster member node senses the environmental physical quantity, and communicates it to its CH. The CH collects the data, aggregates it and transmits it to that node in its communication range which is closest to the BS in the case where the distance of the CH from the BS is less than the defined threshold distance. The inverse case results in direct communication of data if more than one node with the same “distance to BS” exists, then a higher precedence is given to the highest energy carrying node. Apart from the abovementioned, there is a long list of energy-aware routing protocols in WSNs. An updated survey of clustering routing protocols is WSN is given by Liu in [19].

Table 1 gives an abstract view of a comparative analysis of clustering algorithms on various network design and operational parameters. A brief description of parameters used in this table is given below.

- **Node Type**
  Type of deployed nodes according to their configuration.
  ○ *Homogeneous:* all the network nodes have the same configuration (energy, processing power, *etc.*)
  ○ *Heterogeneous:* Nodes in the network have different configurations (energy, transmission range, antenna gain, processing power, *etc.*).
- **Communication to Sink**
  On data collection at the CH, a communication style is chosen to let it reach the BS either through direct communication or through multi-hop communication.
  ○ *Multi Hop:* CH communicates the data to BS through some transient node (CH, or gateway node).
  ○ *Direct Hop*: CH communicates the data to BS without using any transient node.
- **Inter-Cluster Communication Style**
  Communication of data between adjacent clusters for further transmitting it to a BS.
  ○ *CH—CH*: CH communicates the data to its next CH.
  ○ “-”: CH either transmits the data directly to BS or the authors do not mention this routing aspect in the paper at all. Another possibility exists, *i.e.*, a CH does not communicate the data to the next CH but rather it is a gateway node that is selected to transmit the data directly to a BS.
- **Intra-Cluster Communication Style**
  Cluster member node communicates the data to CH either directly or indirectly.
  ○ *Direct*: a CM node communicates sensed or collected data to a CH without using any transient node.
  ○ *Multi Hop*: a CM node communicates sensed or collected data to a CH thorough some transient node.
  ○ “-”: the authors don't discuss intra-cluster communication style in their paper.
- **Cluster Size**
  Size of cluster in network with respect to number of CM nodes.
  ○ *Equal*: No. of CM nodes in network clusters are almost same.
  ○ *Unequal*: No. of CM nodes in network clusters is variable enough to make their size very different from each other.
- **Cluster Design**
  The process of grouping network nodes in clusters is based on some defined parameter.
  ○ *Centralized*: The process of cluster design is controlled directly from a BS.
  ○ *Distributed*: The process of cluster design is distributed. Nodes communicate with each other to do this process.
- **Suitability to Network Size**
  Size of network with respect to deployment area for which the algorithm works efficiently.
  ○ *Small*: Network nodes communicate directly to the BS without any transient node.
  ○ *Large*: Network nodes communicate indirectly to the BS through a number of transient nodes.
  ○ *Medium*: Network nodes communicate indirectly to a BS through one transient node.
- **CH Election Criteria**
  A node elected to head the activities of a cluster is called CH. This election is based on some election parameter.
  ○ *Election Parameter*: the CH is elected based on residual node energy, position of the node, or based on some calculation like ratio of average residual energy of neighbor nodes and residual energy of the node itself.
- Power Adjustment
  Transmission power of node adjustment for communicating its data to destination node.
  ○ *Static*: Nodes' transmission power remains same *i.e.* it is neither increased nor decreased.
  ○ *Dynamic*: Nodes' either increase or decrease their transmission power according to the interaction situation.
- **CH Rotation**
  Role of CH is transferred to a suitable node based on some selection parameter.
  ○ *Rotation Parameter*: CH rotation is performed either on each round, or on a decrease of some node characteristic.
  ○ “-”: the authors don't discuss this aspect in their paper at all.

## 3. Proposed Solution

The routing algorithm for a clustered network is designed either by exploiting the cluster design process or a separate standalone process is initiated for it. In this section, an energy-aware routing strategy is presented for MCDA by exploiting its design process. No large amount of extra broadcasting for forwarding node selection or route establishment is needed since it is pre-set and pre-planned during the cluster design process. This section is divided into three subsections: (1) analysis of MCDA for designing an energy-aware routing algorithm and to highlight the key features for improved performance of the same; (2) introducing the cluster head rotation process and (3) complete routing process.

### 3.1. Analysis of MCDA for EAR

Working of the MCDA in a summarized form has already been presented in Section 2. In continuation of this here we present the analysis for highlighting the parameters that can be used for designing an energy-aware routing algorithm.

Layer 1 nodes broadcast their node density value. The listener nodes of this broadcast among second layer nodes set a forwarding node table having node IDs in the precedence level of their node density. Table 2 shows the sample format of the same for a typical node, say node “w”.

The underlying node first selects the node from the “*Forwarding Node Table*” with highest nodal density. Since the network is homogenous and the energy consumed in cluster design is almost equal in all nodes, the energy delta Δ*E* between the nodes is very small. Hence, all the three nodes of layer 1 (as shown in Table 2) have almost same the energy level. In view of this, the highest degree node is selected first as FN from this Forwarding Node Set (FNS). Higher node degree means more neighbors and so the a big FNS is related to it to share the load of each other and also more nodes to tolerate any expected node faults. More-over, the rotation of FN designation is also easy. For the process of FN rotation, we introduce a two-level threshold strategy. The first level, Load Balancing Threshold, triggers the load balancing process and the second level, Role Transfer Threshold, is for transferring the role of being FN to some other suitable node. We are using almost the same technique in the rotation of CHs in subsequent tiers of our multilayer clustered network architecture. The CHs of the first clustered layer (*i.e.*, second layer of the network) forward their data packets to the selected nodes of the first layer. These collected packets from the second layer nodes are directly sent to the BS by the first layer nodes. In the same way, the CHs of the second clustered layer (third network layer) have decision maker (DM) nodes that are in their communication range. Data is sent to these nodes to further pass it on the way to a BS. The same data packet communication fashion is followed by all the CHs of subsequent layers in order to make the data packets reach the BS.

### 3.2. Cluster Head Rotation

One of the most energy intensive processes in a cluster-based network architecture is cluster head rotation. In this process, the role of being the cluster head is transferred to the most suitable node that has a better selection metric measurement than its competitors. In the homogenous network, each node has the same probability of becoming CH in the first iteration, if the decision metric is a homogenous factor. Let *i* be a node in a network, which has the probability *ρ*_i_ = 1/πr^2^σ to become the cluster head, where σ is the nodal density T_n_/T_a_ where **T**n = total number of nodes in the network and T_a_ = total area of the network. In our case, if T_n_ = 300 and T_a_ = 300 m × 300 m = 90,000 m^2^ then σ = 300/90,000 = 0.0033 nodes/m^2^. Energy depletion, converting the optimality to non-optimality, malfunctions, and entrapment are usually the key causes of CH rotation. To resolve this issue, some algorithms re-initiate the complete clustering process as it was performed the first time, while others randomly select a node among the neighbors of the cluster head and redesign its cluster. Our Energy-efficient Cluster Head Rotation Technique (ECHeaRT) rotates the cluster head designation without disturbing the cluster size and its members. The method for CM nodes to access the CH is also adaptive. It may change from direct hop to multi-hop and *vice versa*. The proposed solution defines a threshold level for the CH energy. In case the minimum number of hops to the BS is considered for the election of cluster head in the cluster head rotation process, then there is maximum possibility that the nodes closest to the base station are elected again and again. Also in a cluster, all the member nodes have almost same hop count to the BS, hence, residual node energy seems to be the most suitable choice as a decision metric for the cluster head in this underlying scenario, so in order to achieve the distinguished energy awareness in cluster head rotation by ECHeaRT, threshold levels for CH energy are exploited in two steps:

(i) Load Balancing Threshold (LBT); balancing the load on CH with the Backup Forwarding Node (BFN) when the energy level reaches almost 50% of the initial energy. This initial energy is noted when a node is designated as CH.
(ii) Role Transfer Threshold (RTT); transferring the role of being CH from an existing CH to the new CH (previously working as BFN) when the energy level approaches about 20% of the initial energy. This initial energy is noted when the node is designated as CH.

**Figure 2.**
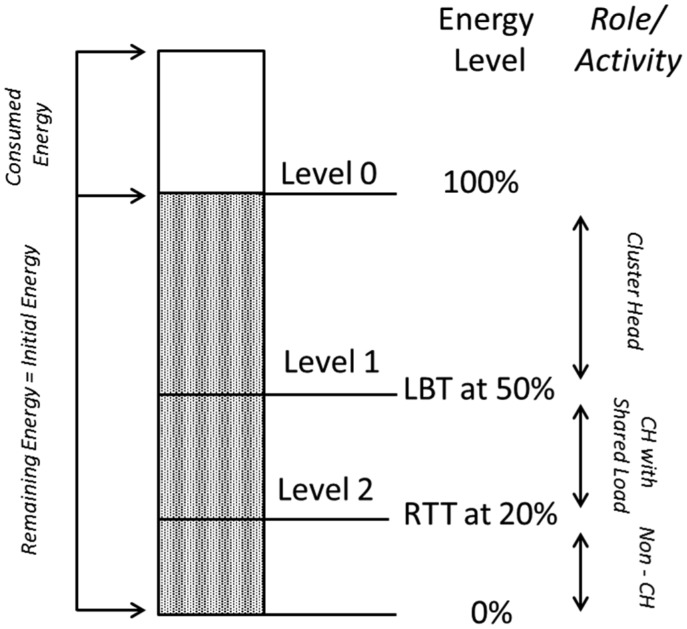
Switching of a CH to different roles based on its energy threshold levels.

A depiction of the process for switching a CH in different roles based on its energy threshold levels is given in Figure 2. On reaching *E_c_* = *E_i_/*2 (*i.e.*, the current energy of a node that is equal to half of the initial energy of that node) *i.e.*, LBT status, the switching function triggers to change the role of “CH” to “CH with shared load”. A CH Rotation message *Msg* (*CHR_init_*) is initiated from the CH towards CMs to get their energy levels. Since the decision metric for the selection of the next CH is being the highest energy carrying node, on the basis of the collected data the next potential cluster head is decided by the existing CH, *i.e.*, termed Backup Forwarding Node, for now until a complete assignment of the role of CH is accomplished. The decision is communicated to the selected node and the acknowledgement is received. The cluster member nodes that receive this acknowledgement directly from the newly decided CH (BFN) send their data packets to it while the other member nodes of the same cluster still keep on communicating their data to the existing cluster head.

On reaching *E_c_* = *E_i_*/5, *i.e.*, 20% of the initial energy of the node (RTT level), the existing CH broadcasts a message to its member nodes to communicate the info of “role transfer to BFN”. In this scenario, two cases exist:

*Case I*: If all CM nodes have direct access to the CH.
  In this case, the notification of the existing CH regarding its role transfer to BFN is directly listened by all CM nodes. These CM nodes set their CH field with the newly designated CH and later on communicate their data to it for aggregation.
*Case II*: If some nodes have indirect (multi-hop) access to CH and other have direct (single-hop) access.
  Since, ECHeaRT offers both direct and multi-hop access of CM nodes to the CH. for the multi-hop communication, the intermediate node between the transmitting CM and the targeted CH communicate the new CH decision to its linked CMs. This node then sets its CH field to this newly designated CH and communicates with it. The other nodes which have direct access to existing CH listen to the role transfer decision directly and set their CH field to this new one. With this, the existing CH is demoted to a non-CH node role, *i.e.*, CM and BFN are promoted to CH. In this new setup, the cluster size and its cluster members remain the same. The only consumption of energy is in the CH role transfer and in communicating this decision to the cluster members.

### 3.3. EAR4MCDA: Energy Aware Routing Strategy (EAR) for Multilayer Cluster Designing Algorithm

In the proposed Energy Aware Routing Strategy scheme, the status of a node is switched between different roles due to the rotation of “Forwarding Node” designation during the inter-cluster routing process. This is performed to save the network from partitioning and to prolong the network lifetime. In a sensor network, strategies which utilize to the maximum network nodes (also called network utilization) with maximum delay in the death of the first node are appreciated. This shows balanced behavior of the algorithm over network nodes and less danger of creating a void due to network partitioning as well. Rotation of CH designation is one of the initial steps towards it. The roles of switching in the four various designations are Decision Maker Node (*DM_Node_*), *FN*, *BFN* and Non- Forwarding Node (*NFN*). The subsequent paragraphs briefly explain these roles:

#### 3.3.1. Decision Maker Node

The first elected FN by the CH is always from the list of *DM_Node_* and satisfies the following condition:

$$DM_{\text{Node}}\to FN=\text{Cnt}r_{n\left(i\right)}>\text{Cnt}r_{n\left(j\right)}\forall_{j}\text{and}\left|D(N_{i}-N_{j)}\right|\leq r_{i}\forall_{j}\text{Where}j=1,2,\ldots ,n$$

*i.e.*, the node having highest neighbor count, *Cntr_n_*_(_*_i_*_)_ among its competitors (nodes in its communication range, *r_i_*) is promoted from *DM_Node_* to FN, while *DM_Node_* are selected from FNS listed at CH of layer 2.

#### 3.3.2. Forwarding Node

The node that is elected to pass on the received packet toward the BS is called forwarding node. Nodes with any of the roles from among BFN, NFN or *DM_Node_* can be upgraded to FN after winning some defined competition at various levels of operation.

*Case I*: *DM_Node_* → FN*Case II*: *BFN* → *FN**Case III*: *NFN* → *BFN* → *FN*

#### 3.3.3. Backup Forwarding Node

This node shares the FN load when its energy reaches the LBT threshold. The BFN is later upgraded to FN once its accompanying FN is degraded to NFN. To start, the node which satisfies the following condition is selected as BFN:

$$\text{BFN}\to FN=E\left(N_{i}\right)>E\left(N_{j}\right)\forall_{j}\text{and}\left|D(N_{i}-N_{j)}\right|\leq r_{i}\forall_{j}\text{Where}j=1,2,\ldots ,n$$

*i.e.*, nodes having highest energy among their competitors are promoted from BFN to FN. Promotion of NFN to BFN follows a similar energy priority rule.

#### 3.3.4. Non-Forwarding Node

The *DM_Node_* which has once acted as FN and finished its turn of being FN is termed as NFN. Also the node which is neither a FN nor BFN or *DM_Node_* is given the name of NFN. The promotion of NFN to BFN only arises when CH does not have any *DM_Node_* unelected as FN in its FN list. NFN is upgraded to BFN and later to FN on reaching the specified condition:

NFN→BFN→FN

Step 1: $$\text{NFN}\to \text{BFN}=E\left(N_{i}\right)>E\left(N_{j}\right)\forall_{j}\text{and}\left|D(N_{i}-N_{j)}\right|\leq r_{i}\forall_{j}\text{Where}j=1,2,\ldots ,n$$
  *i.e.*, the node having the highest energy among its competitors is promoted from NFN to BFN.
Step 2: $$\text{BFN}\to FN=E\left(N_{i}\right)>E\left(N_{j}\right)\forall_{j}\text{and}\left|D(N_{i}-N_{j)}\right|\leq r_{i}\forall_{j}\text{Where}j=1,2,\ldots ,n$$

**Figure 3.**
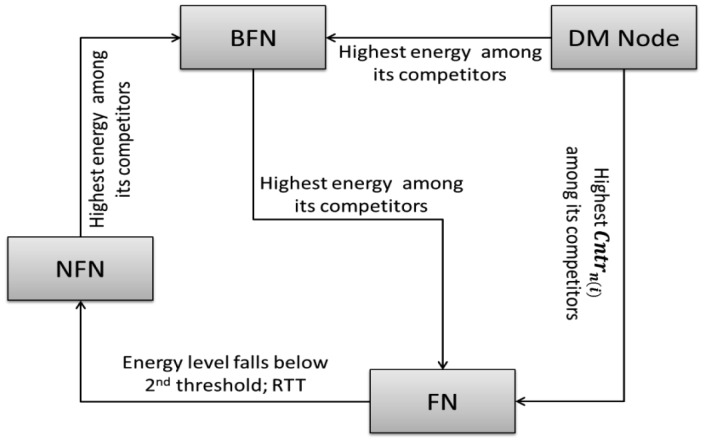
Different roles of network nodes in rotation of forwarding node.

*i.e.*, the node having the highest energy among its competitors is promoted from BFN to FN. Figure 3 summarizes these roles, their rotation and a brief description of their selection criteria.

**Figure 4.**
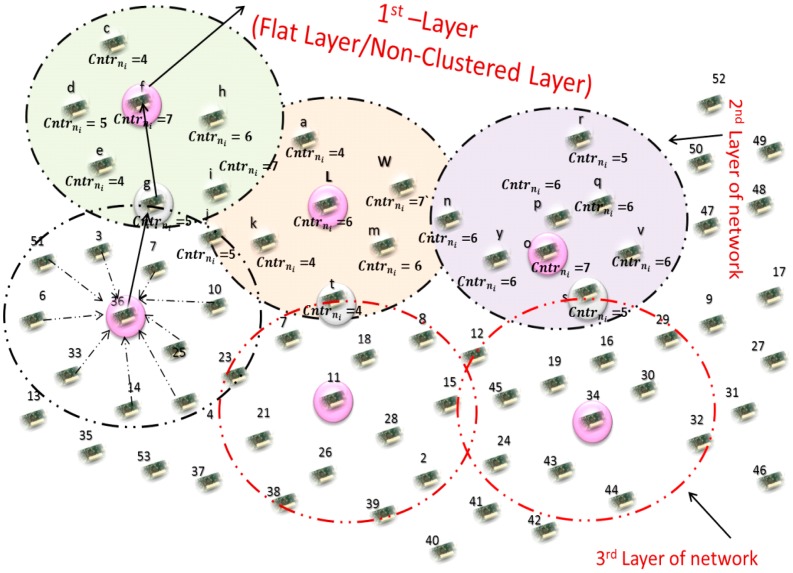
Intra-cluster and inter-cluster routing in MCDA network.

In view of above discussion, we can briefly summarize the working of EAR as follows: cluster member nodes communicate their collected data to the cluster head. Like all other nodes of the underlying layer, the CH under discussion also has a forwarding node table that has the node IDs of its decision maker nodes (*DM_Node_*) in the preceeding level of their node degree. Nodes at the top in the list are selected as the forwarding node (FN). If this forwarding node is not the CH then it directs the data to its CH. In the other case the CH sends the collected data to the “node with highest node degree carrying value” from its neighbor table. This process continues until data is collected at the BS. Figure 4 depicts these intra-cluster and inter-cluster routing processes in a more understandable form.

**Figure 5.**
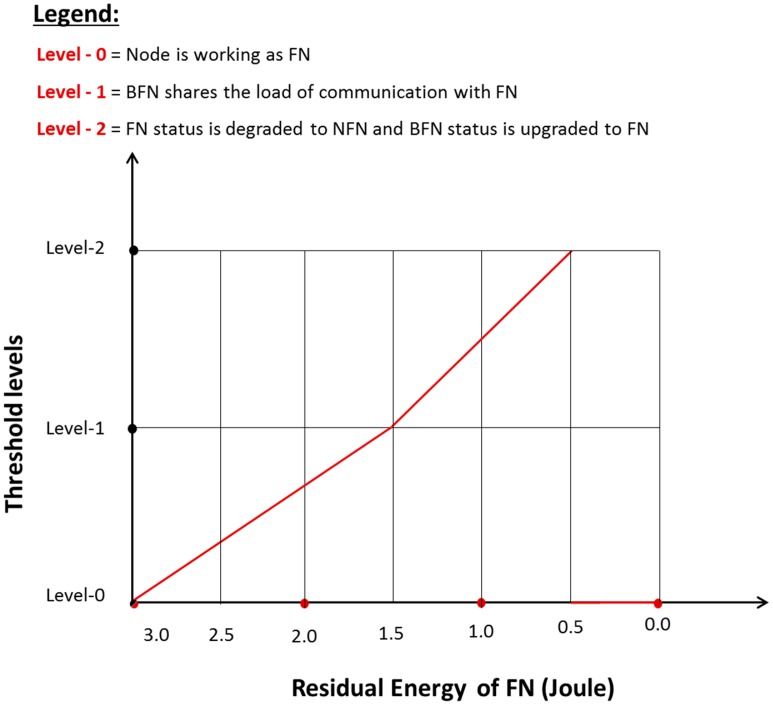
Operation of threshold levels in forwarding node rotation.

Rotation of FN follows the same process as is discussed for the CH rotation process. Two threshold levels are defined for the rotation of FN, as depicted in Figure 5.

At level 0, the node is working as FN until level 1 where its energy is decreased to 50% of the initial energy. On approaching level 1, FN broadcasts a *Load_Sharing_Msg message*. Recipient nodes of this message decrease the transmission load towards this node to half, *i.e.*, alternate packets are transmitted to it. The remaining half transmission is directed towards the next highest node density carrying node that is called a BFN. On approaching level 2, *i.e.*, about 20% of the initial energy, transmission is totally directed towards the BFN since its status has already been upgraded to FN and the previous FN is demoted back to being a simple network node. This same process is used for forwarding node rotation throughout the network.

## 4. Comparative Analysis of Proposed Solution with EADUC and TLPER

Though the presented ideas related to cluster designing, cluster head selection, node affiliation to CH as CM, forwarding node selection, inter- and intra-cluster routing and cluster head rotation. These are all combined under the name Energy Aware Routing for Multilayer Cluster Designing Architecture (EAR4MCDA), there are close similarities with some existing techniques in one functional aspect or the other. For this very reason, we have selected TLPER and EADUC for the performance evaluation comparison of our proposed technique. We have given sufficient details of the workings of both the algorithms in the literature survey section. The authors of TLPER compared its performance with LEACH on energy consumption per node, cluster head, and assistant cluster head, network utilization, and load balance effect on energy consumption, where it outperforms its competitor LEACH. The performance of EADUC on network lifetime is compared with EEUC, LEACH-M, LEACH and HEED and the authors found it better in this aspect in comparison to its competitors. In this section, a comprehensive discussion of a comparative analysis of EAR4MCDA with state-of-the-art related algorithms—TLPER and EADUC—is provided, based on the following performance metrics:

- Energy consumption in cluster design
  ○ Before the start of the operational phase of the network, it needs to be setup for it. We have measured the energy that is consumed in designing the clusters, *i.e.*, making the network ready for the operational phase.
- Energy consumption in forwarding node selection
  ○ The current node needs to select a node among its neighbors for pushing the data ahead to the BS. That selected node is termed the forwarding node. We have measured the energy consumption that is used in information communication for the selection of forwarding nodes.
- Energy consumption for transmitting one packet from source to destination
  ○ This energy measurement is directly related to the routing part of the algorithm. We have calculated the energy that is consumed for dissemination of generated data from the far distant network node until the BS. Other aspects of energy consumption are also included in it, e.g., forwarding node selection, *etc.*
- No. of Designed Clusters
  ○ Total numbers of clusters that are designed in the network.
- Average number of hops from end to end
  ○ The nodes are deployed randomly and the network adopts the shape of a Voronoi diagram. This results in variable end to end (E2E) distances and therefore a variable E2E number of hops. We have taken the average of these E2E number of hops as part of the performance metrics list.
- Number of packets produced in the first 60 s after cluster formation
  ○ During the network operation phase, we have measured the number of data packets produced in first 60 s. This performance metric is used with the generated throughput in the first 300 s.
- Throughput in the first 300 s
  ○ Successful delivery of data packets is also taken as another performance evaluation parameter. We have measured the throughput in the first 300 s of the network operation phase.
- Energy consumption for total throughput in the first 300 s
  ○ We have calculated the energy consumption for successful reception of data packets at the BS in the first 300 s of the operational phase.
- Energy consumption for cluster head rotation
  ○ Energy consumption during CH rotation encompasses all the activities that are undertaken from the time of CH rotation until this designation is completely transferred to a new node.

We have run the simulation a number of times in a network of 500 nodes in an area of 500 m × 500 m. The succeeding results from Figures 6, 7, 8, 9, 10, 11, 12, 13, 14 and 15 represent the average of the number of executions. Simulation parameters are given in Table 3.

**Figure 6.**
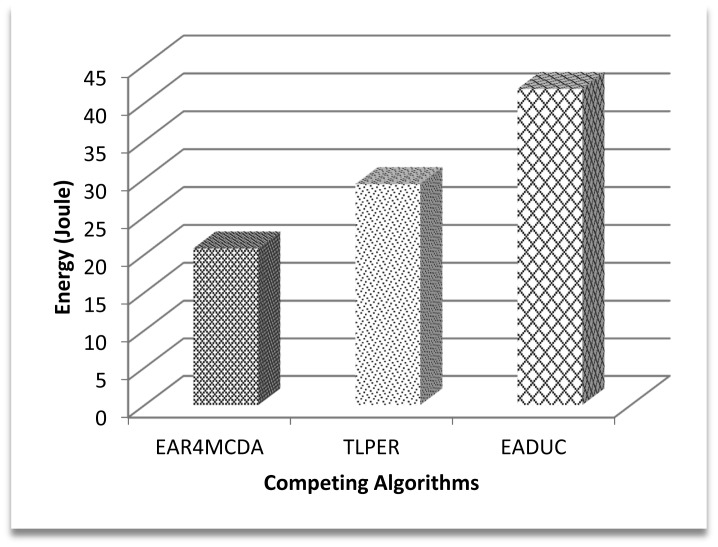
Total energy consumption in cluster design.

The contribution of node energy in the data routing process starts from the cluster design process and ends with the data approaching the sink. Figure 6 shows that the cluster design part is an indispensable phase in considering energy-aware routing. In a network of 500 nodes in the area of 500 m × 500 m, about 41 J of energy is consumed just in designing the clustered network while implementing the EADUC algorithm. This consideration is reflected more when comparing the energy consumption of the three competing algorithms during the cluster designing process (Figure 6).

**Figure 7.**
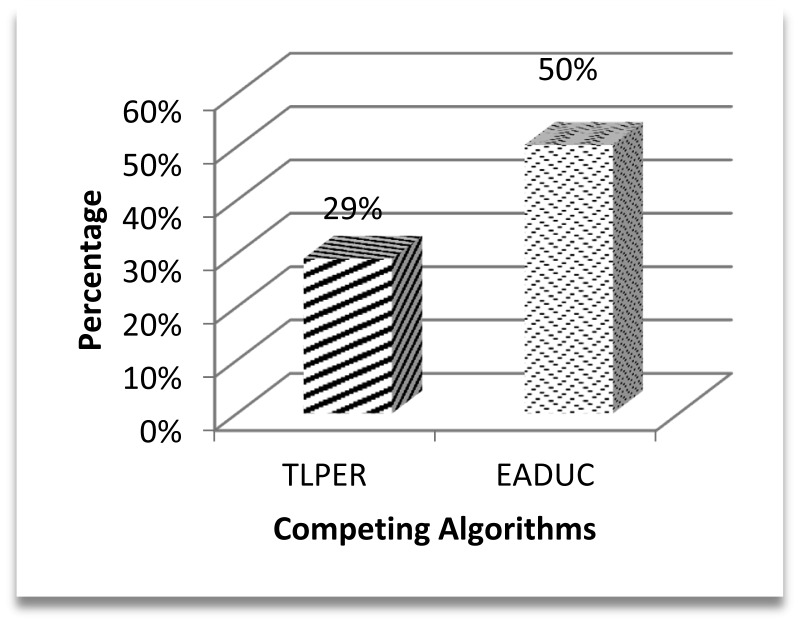
Performance efficiency of EAR4MCDA over competing algorithms in total energy consumption.

Figure 7 demonstrates this effect with 29% better performance of the proposed algorithm over TLPER and 50% over EADUC. Intra- and inter-cluster routing makes it possible for this sensed data to reach the destination to end the routing. The energy efficient selection of forwarding nodes plays a vital role in the overall energy-aware routing process, as this selection decision has an effect on path length, node congestion, throughput and consequently energy conservation.

**Figure 8.**
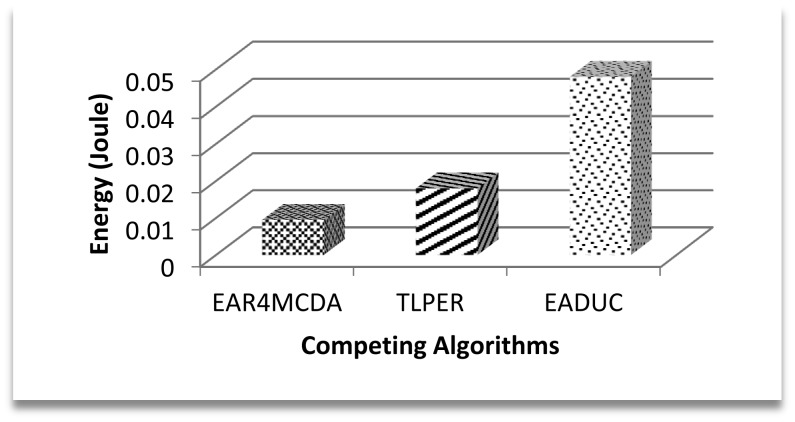
Average energy consumption in forwarding node selection.

In this underlying research article, we have evaluated the selection of FN with respect to energy consumption only. Figure 8 shows the competitive energy consumption factor in forwarding the data to the FN. EAR4MCDA presents the innovative idea of having a list of selected FNs (three in advance) at each node during the cluster design process, so no extra energy conservation results at this point, while in case of TLPER and EADUC, each node that intends to forward the packet needs to collect decision metric information from its neighbor nodes. From this FNS, the most suitable node having the highest decision metric precedence is selected (vicinity head in the case of TLPER). All this process really squeezes the nodes' energy, which deteriorates the overall performance of the algorithm. This is ultimately reflected in the form of higher energy consumption. In this regard, the performance of EAR4MCDA is 39% and 80% better than TLPER and EADUC, respectively. On comparing the performance of TLPER and EADUC, we come up with the result of 68% outperformance of the former over the latter.

In TLPER, FNS may comprise the next CH, assistant CH and BS. The current node selects any one among them that is within reach and communicates the data to it, while in case of EADUC, the current CH disseminates a query message among its neighbor nodes and collects their distance to the BS and their energies. If more than one node has the same proximity to BS then their residual energy is used to break this tie.

**Figure 9.**
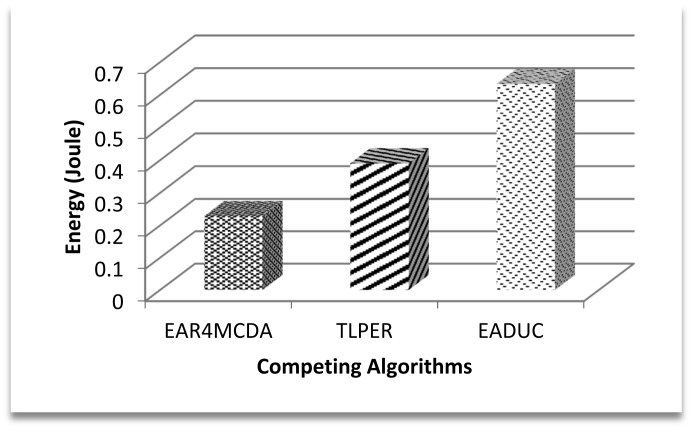
Average energy consumption for transmitting one packet from end to end.

The effect of this high energy consumption in FN selection makes the overall communication expensive, since the FN in the path makes up a route for packet dissemination from source to sink. Figure 9 illustrates this point. Expensive FN selection in EADUC results in more costly communication of a data packet from end to end. In the graph, the resulting value bar for EAR4MCDA is far smaller than that of EADUC due to the aforementioned reason, though the path length and other factors also matter, so to make a fair comparison, these calculations are taken for the same path length.

**Figure 10.**
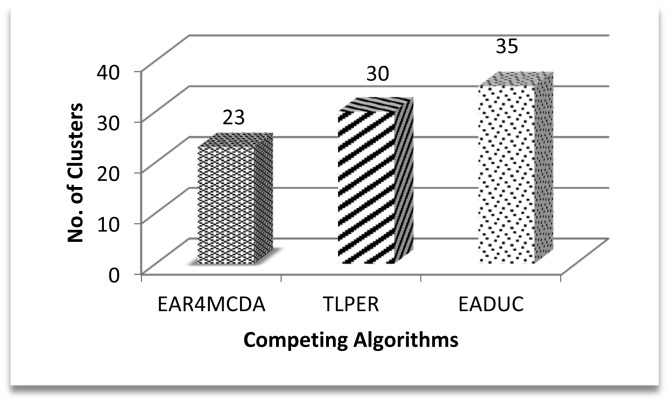
No. of designed clusters.

Network size, deployment area and node deployment style are almost the same, so the number of designed clusters must certainly be the same but the different cluster design strategies of these algorithms make the difference. In EAR4MCDA, the first layer, which covers about a 50 m–70 m area, does not have clusters—that's why it is termed a flat layer. In the remaining area, the cluster size is almost the same. In the case of TLPER, the proximity of nodes to the CH that is based on the Received Signal Strength Indicator (RSSI) is what defines the cluster size, while in case of EADUC, cluster size is directly related to proximity to the BS. The lesser the distance of a cluster to the BS, the smaller is its size and *vice versa*. Based on the above discussion, the higher number of clusters in EADUC compared to TLPER and EAR4MCDA is logical. This comparison is depicted in Figure 10.

**Figure 11.**
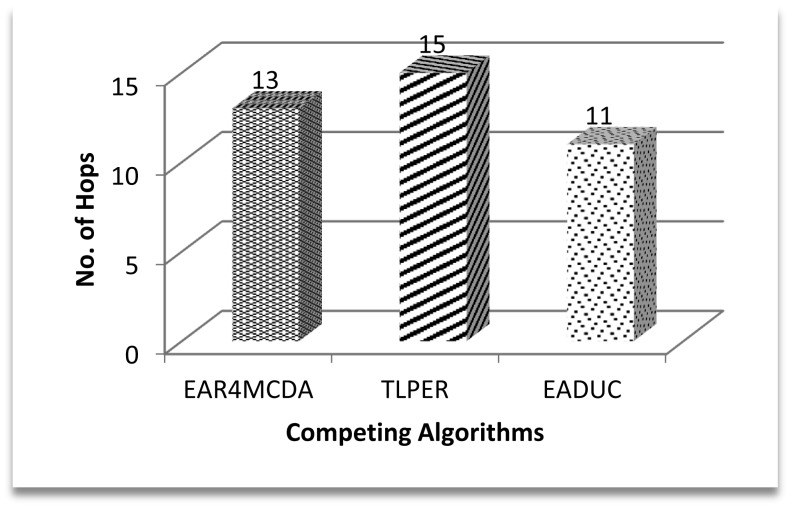
Average no. of hops from end to end.

In extension to the previous discussion, it can be construed that a bigger number of clusters may mean more end to end hops, but this again depends upon the inter- and intra-cluster routing strategies. Aggregated data at the CH is sent to any next hop neighbor node which is closest to the BS. While in TPLER, inter- and in some cases intra-cluster communication is multi-hop, so there are more hops in any related communication path in the case of EADUC and EAR4MCDA. Figure 11 illustrates these points.

**Figure 12.**
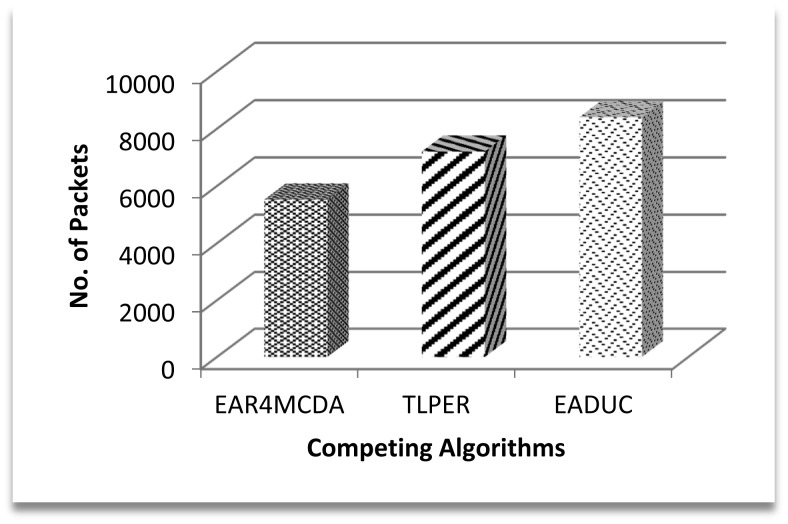
No. of packets produced in the first 60 s after cluster formation.

Coming back to Figure 9 and correlating its result with Figures 10 and 11, we can intuit that forwarding node selection has its vital role in energy conservation and thus in energy-aware routing. Throughput is another important scale of measuring the efficiency of routing protocol. A higher production packet number at the source node does not mean an increased throughput. Moving one step back to this point, more clusters may mean more packet production numbers. Let there be a scenario of 100 nodes. If there are five clusters and on the average each cluster may comprise of 20 nodes, the estimated waiting time for a node to communicate its data to a cluster head is equal to 19 or 20 time slots under the TDMA MAC protocol. If the data rate is four packets per second then at least 20/4 = 5 s are required to complete one cycle. During this time of 5 s, 20 packets are expected to be produced by keeping all other things constant. Since clusters' work in parallel and not in series, the number of packets that are produced in five clusters is (5 × 20) =100 packets. In another comparative scenario of 10 clusters with 10 nodes in each cluster with the same data rate and same conditions as in previous scenario, 2.5 s are needed to complete one cycle with the production of 10 packets and in 5 s 20 packets are formed in the cluster, so a maximum of 200 packets (20 × 10) are produced in an ideal case in this scenario. Based on these two scenarios, the higher number of packets produced in EADUC compared to TLPER and EAR4MCDA is logical (Figure 12).

**Figure 13.**
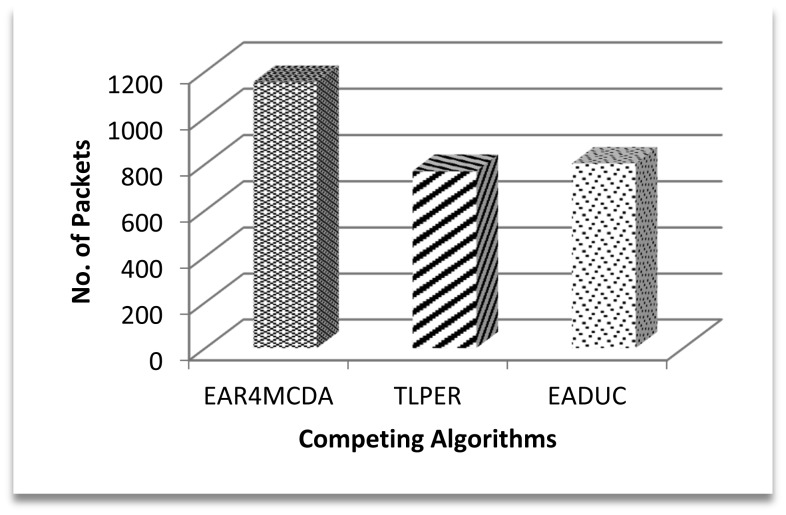
Throughput in first 300 s.

However, more packet production does not mean better throughput. Figure 13 plots the results of throughput in the first 300 s. The graph represents the superiority of EAR4MCDA over TLPER and EADUC with a performance efficiency of 33% and 30%, respectively. The very apparent reason for this outperformance lies more in FN selection in a complex way that gives rise to longer queues in nodes' buffers and hence more node delay leading to increased end to end delay. This may further result in more packet losses and thus the decreased throughput (Figure 13).

**Figure 14.**
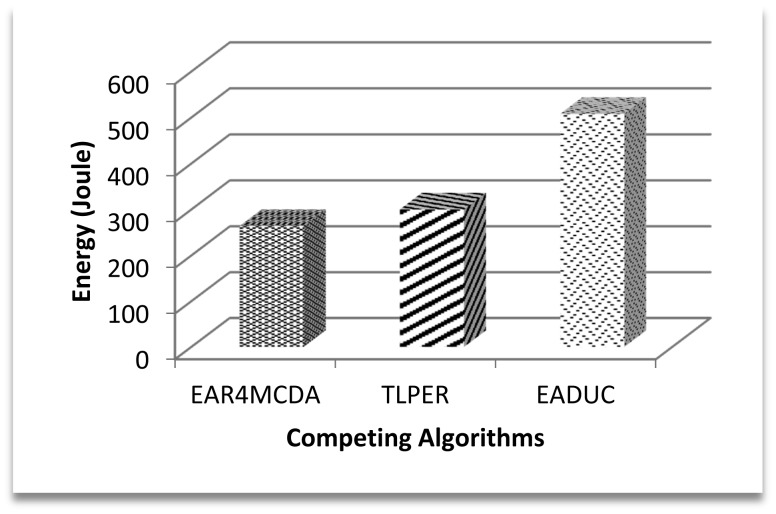
Energy consumption for total throughput in first 300 s.

This reflects overall poor network performance with non-energy aware routing. Figure 14 shows the energy consumption for the total throughput for the first 300 s.

**Figure 15.**
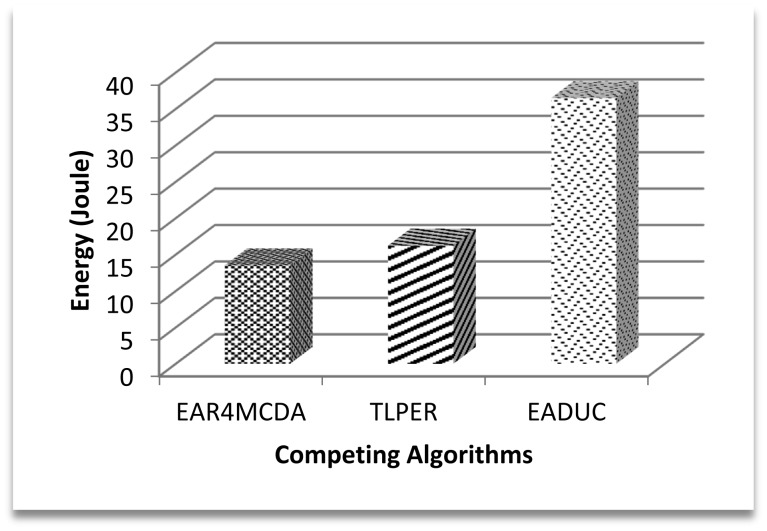
Energy consumption for cluster head rotation.

We have also evaluated the performance of the competing algorithms on energy consumption for the CH rotation parameter that is depicted in Figure 15. EADUC does a completely new design of clusters in each round that increases its energy consumption value during the CH rotation process while TLPER transfers the CH role to the already selected assistant CH node. Almost same procedure is followed in EAR4MCDA. This provides a clear differentiation between the energy consumption performances of EADUC with other two. EAR4MCDA and TLPER consume almost same energy.

## 5. Conclusions

Network performance is the cumulative effort of a number of integral and integrated steps. EAR4MCDA was an endeavor to ameliorate the network performance from an energy consumption aspect in the routing process. The architecture of MCDA was exploited for FN selection, inter- and intra-cluster routing, CH rotation and all the way to final data delivery to make this overall process energy-aware. Empirical results intuit that careful selection of FN leads to energy efficient intra-cluster and inter-cluster routing that combine to provide energy-aware routing. It is also construed from the results that though more clusters may produce more numbers of packets, other favorable factors are at play which result in improved throughput. Otherwise, it is just a burden on the network and results in increased congestion, more packet drop ratio, more end to end delays and hence more energy consumption is needed to alleviate these issues and maintain the network performance. It is also deduced from the experiments that cluster head rotation in each round is not a sensible choice to implement, but rather letting the CH work until it consumes a specified amount of its energy, and then rotates its designation to some other suitable node, is a better option.

## Figures and Tables

**Table 1.** Comparative analysis of clustering algorithm on various network design and operational parameters.

	[5]	[10]	[18]	[20]	[11]
**Node Type**	Homogenous	Homogenous	Both	Homogeneous	Homogenous
**Comm. to Sink**	**-**	Multi-hop	Multi-hop	Multi-Hop	Multi-Hop
**Cluster Size**	Near Equal	Near Equal	Unequal	Unequal	Unequal
**Cluster Designing**	Hybrid	Centralized	Distributed	Distributed	Distributed
**Suitability to Network Size**	Large	Small	Large	Large	Large
**CH Election Criteria**	Node Density	Node Energy	Ratio of Avg. RE of neighbor nodes and RE of Node itself	Node Degree	Node Energy
**Inter-Cluster Comm. Style**	**-**	CH to CH	CH-CH	CH to CH	CH-CH
**Intra-Cluster Comm. Style**	**-**	Direct	Direct	Direct	Direct
**Power Adjustment**	Static	Dynamic	Dynamic	Dynamic	Dynamic
**CH Rotation**	**-**	On each round	On each round	On decrease in Node density	On each round

**Table 2.** Forwarding Node Table at node “w”.

**Decision Maker Candidate Node ID from Layer 1 Nodes**	**Node Density**
Y	7
U	7
Z	8

**Table 3.** Simulation parameters.

**Parameter**	**Description**
Routing Protocols	EADUC, TLPER, EAR4MCDA (Proposed)
Simulation Area	500 m × 500 m
Simulator	NS 2.31
Data Rate	4 Packets/S
TCP/IP Layer	Network Layer
Node to Node Distance	Random
Node Type	Homogenous
No. of Nodes	500
Propagation Model	Two ray ground
Initial Energy of Node	3 J
